# Shaping the future of multiple myeloma with artificial intelligence and digital twins: from concept to clinic

**DOI:** 10.3389/fdgth.2026.1771531

**Published:** 2026-03-18

**Authors:** Cindy H. Lee, Yang Zhang, Barbara J. McClure, Angelina Yong, Hamish S. Scott, Chung Hoow Kok

**Affiliations:** 1 Department of Haematology, Royal Adelaide Hospital, Central Adelaide Local Health Network, Adelaide, SA, Australia; 2 Faculty of Health and Medical Sciences, Adelaide University, Adelaide, SA, Australia; 3 Department of Genetics and Molecular Pathology, SA Pathology, Adelaide, SA, Australia; 4 School of Pharmacy and Biomedical Science, College of Health, Adelaide University, Adelaide, SA, Australia; 5 Centre for Cancer Biology, an alliance between SA Pathology and Adelaide University, Adelaide, SA, Australia

**Keywords:** myeloma, functional high risk, cytogenetics, multi-omics integration, AI, digital twin, risk prediction, risk stratification

## Abstract

Multiple myeloma (MM) is an incurable hematological malignancy with significant clinical and biological heterogeneity. Despite development and refinement of numerous prognostic models for MM, challenges with accurate and reliable risk stratification remain, highlighted by unexpected, early relapse or progression of disease in patients termed functional high-risk (FHR). To improve decision-making and optimise outcome, there is an unmet need for precise identification of high-risk (HR) patients, to enable tailored therapeutic strategies. With a complex and rapidly evolving treatment landscape, artificial intelligence (AI) and digital twin (DT) technology have emerged as potential tools for personalized medicine in MM. Through the integration and analysis of large data generated in clinical trials, registries and real-world cohorts, AI can inform therapy selection by creating advanced predictive models. DT, virtual patient-specific disease replicas, act as a dynamic, bidirectional bridge between real-world clinical data and computational simulations. Continuous acquisition of patient data, synchronized with DTs through AI-driven architectures, facilitates iterative risk recalibration. This ensures the virtual models accurately reflect evolving disease biology and treatment response. This review provides an overview of current and emerging risk stratification in MM, including genomic-based definitions of HR disease and the concept of FHR MM. We described the role, limitations and controversies of AI and DT in refining risk assessment, their predictive capacity for outcomes and therapy selection. Finally, we provide perspectives on the future of AI application in MM.

## Introduction

Multiple myeloma (MM) is a hematological malignancy arising from malignant transformation of plasma cells, with an annual incidence of 188,000 new cases globally (1). Despite advances in its treatment landscape with improved access to novel and immune based therapies, MM remains incurable, largely due to its clinical and biological heterogeneity precluding precise risk stratification to inform selection of therapy (2–5). Suboptimal outcomes, defined by early disease progression and treatment refractoriness, continue to be observed in subsets of patients, with an estimated 20% of newly diagnosed MM (NDMM) patients invariably relapsing within 24 months from diagnosis (6).

Extensive datasets from clinical studies, registries and real-world cohorts, together with advances in genomic profiling and multi-omics technologies, have led to increasingly refined and more sophisticated prognostic models for MM. However, significant limitations remain, including poor specificity, inter-classification discordance, and indifference to multi-hit disease. These are further compounded by the rapid pace of development of new MM therapies, limiting their relevance and real-time application in clinical practice (7).

Digital health technologies, particularly artificial intelligence (AI) (8) and digital twin (DT) (9) models, can play a critical role in addressing these challenges. AI approaches, incorporating machine learning and deep learning (8), can integrate the massive volume of patient-derived information (multi-omics, imaging, and longitudinal clinical data), to generate real-time, data-driven clinical workflow. DTs, which are customized, virtual replicas of patients, enable simulation of disease progression and real-time therapeutic responses (9).

In this review, we summarize current and evolving risk stratification models in MM, focusing on the genomic-based definitions of high-risk (HR) disease and the entity of functional high-risk (FHR) MM. We describe the role of AI and DT in refining risk assessment of this complex disease, their capacity in predicting survival and informing decision-making, as well as their limitations and controversies.

## Evolution of risk stratification systems in MM

Older systems such as the Durie–Salmon Staging (DSS, 1975) focused on tumor burden and failed to reflect biological complexity (10). As our understanding of the heterogeneity of MM and the prognostic relevance of its genomic abnormalities increase, risk stratification systems have evolved. The International Staging System (ISS, 2005) (11), and subsequent revisions (R-ISS, 2015 and R2-ISS, 2022) (12, 13) incorporate different parameters but only partially captured biologic heterogeneity. The Mayo Additive Staging System (MASS, 2022), an additive 5-factor system, identified subgroups with genomic abnormalities, including del(17p), *TP53* mutation, biallelic del(1p32), and 1q21 gain, which correlated with aggressive disease and poor survival (14).

In 2025, the International Myeloma Working Group (IMWG) and International Myeloma Society further updated risk stratification, defining HR MM as the presence of any of the following: (1) deletion 17p in >20% of plasma cells, (2) a *TP53* mutation, (3) biallelic deletion of 1p32, or (4) the co-existence of any 2 intermediate-risk abnormalities - t(4;14), t(14;16), gain of 1q, or monoallelic deletion of 1p32, together with elevated beta-2-microglobulin in the setting of preserved renal function (15). Current validated prognostic models and risk stratification systems in MM are summarised in Table 1, these include biomarkers of disease burden, key cytogenetics, interphase FISH and molecular abnormalities.

**Table 1:** Established and emerging risk stratification systems in newly diagnosed multiple myeloma (NDMM).

Model (Year)	Clinical Parameters	High-Risk Cytogenetic Abnormalities (HRCA)	Definition of High-Risk
DSS (1975) (10)	Hb, LDH, calcium, serum paraprotein, Xray (Bony disease), BJP	N/A	Stage III = Hb < 8.5 g/dL; serum calcium >12 mg/dL; IgG paraprotein >7 g/dL or IgA > 5 g/dL; BJP >12 g/24 h. >2 lytic lesions on Xrays
ISS (2005) (11)	Albumin B2M	N/A	Stage III=B2M > 5.5 mg/L
R-ISS (2015) (12)	ISS stage (B2M, Albumin), LDH	del(17p), t(4;14), t(14;16)	ISS III + LDH > ULN or HRCA present
R2-ISS (2022) (13)	ISS Stage: II =1 pt III =1.5 pts; LDH > ULN =1 pt	del(17p) = 1.5 pts t(4;14) = 1 pt gain/amp(1q) = 0.5 pts	Composite score of 3–5 points
MASS (2022) (14)	LDH > ULN, B2M Albumin	del(17p); t(4;14); t(14;16); t(14;20); gain/amp(1q)	1 point for each risk factor High-risk if Composite score ≥2
IMWG (2025) (15)	B2M Albumin	del(17p), t(4;14), t(14;16), t(14;20), gain/amp(1q), del(1p), *TP53* mutation	High-risk if any of: 1. del(17p) and/or *TP53* mutation 2. biallelic del(1p32) 3. t(4;14), t(14;16), or t(14;20) co-occurring with 1q21 gain or monoallelic del(1p32) 4. monoallelic del(1p32) co-occurring with 1q21 gain 5. elevated B2M with normal renal function
FUTURE MODELS	EMD, primary PCL, ctDNA, MRD, frailty, immune profiling	High-risk GEP, mutational signatures, High-risk biomarkers derived from multi-omics	High-risk if these are present

DSS, Durie–Salmon staging system; ISS, International Staging System; R-ISS, Revised International Staging System; R2-ISS, Second Revision of the International Staging System; IMWG, International Myeloma Working Group; MASS, Mayo Additive Staging System; NDMM, newly diagnosed multiple myeloma; LDH, lactate dehydrogenase; ULN, upper limit of normal; B2M, β2-microglobulin; Hb, hemoglobin; BJP, Bence Jones protein; HRCA, high-risk cytogenetic abnormalities; GEP, gene expression profiling. Cytogenetic notation: del, deletion; amp, amplification; gain, copy-number gain; t, translocation; EMD, extramedullary disease; PCL, plasma cell leukemia; ctDNA, circulating tumor cells DNA; MRD, measurable residual disease; N/A, not available.

Further refinement of these models are anticipated as additional biological and clinical determinants of adverse outcomes are identified. Collaborative effects are ongoing to validate tumor-specific factors, including plasma cell leukemia, circulating tumor cells and extramedullary disease (16–18). Host-related factors (biological frailty and immune fitness) (19, 20), HR gene expression profiling signatures [e.g., SKY92 (21), UAMS70 (22)], and overexpression of poor prognosis genes such as *DSG2* (23) and *PHF19* (24) are also emergent contributors to risks.

Measurable residual disease (MRD) has likewise become a key prognostic marker in MM (25). MRD is currently assessed using methodologies which interrogate distinct biological compartments. Bone marrow-based techniques include next-generation flow cytometry (NGF) and next-generation sequencing (NGS) (26, 27). NGF identifies malignant plasma cells using standardised multiparameter panels, whereas NGS tracks patient-specific immunoglobulin gene rearrangements, enabling molecular clonal tracking over time. Imaging modalities with FDG PET/CT and whole-body diffusion-weighted MRI, provide functional assessment of focal and extramedullary disease that may be missed by single-site bone marrow sampling, capturing spatial disease (28).

Each modality of MRD assessment is associated with inherent strengths and limitations (27). NGF is rapid but operator-dependent; NGS provides deep sensitivity but requires baseline clonotype identification; imaging captures spatial disease distribution but with lower molecular resolution (28). Collectively, this underscores the need for integrative interpretation of data rather than reliance on a single platform.

Achievement of MRD negativity, defined by the IMWG as the absence of clonal plasma cells at a sensitivity of 10^−^⁵ on bone marrow assessment (29), disappearance of all previously increased metabolic tracer uptake (30), and the durability of MRD negativity, are consistently associated with superior survival across treatment modalities and patient subgroups (31). Incorporation of MRD assessment into risk stratification for MM allows for dynamic, personalized assessment which better informs individual patient outcomes, and can help guide the intensity of ongoing therapy (32).

## Functional high-risk MM: a subgroup of MM without identified high-risk features

Functional high-risk (FHR) MM is characterized by unexpected, early relapse of MM despite the absence of validated baseline molecular or clinical HR features (33). Emerging evidence suggests that extramedullary disease, circulating plasma cells, HR gene expression signatures, and patient frailty are key covariates underpinning this phenotype (33).

Broader access to contemporary induction regimens, incorporating immunomodulators, proteasome inhibitors, anti-CD38 antibodies, autologous stem cell transplantation (ASCT), and maintenance therapy has substantially improved outcomes for most patients. However, a proportion still relapse early despite lacking recognized HR markers (34). This discordance raises concerns about the adequacy of current risk stratification models, highlighting the need to identify additional drivers of aggressive disease biology.

With expanding therapeutic options and increasing clinical trial availability, both the incidence and operational definition of FHR MM have evolved. Drawing on four major real-world and clinical trial datasets, the recent European Myeloma Network (EMN) Consensus Statement recommends defining FHR MM as relapse within 18 months of initiating any frontline therapy (35). This time-based definition captures patients irrespective of age or transplant eligibility, and provides a harmonized framework for interpreting subgroup analyses and designing trials focused on this HR population.

Using the 18-month threshold, approximately 20% of newly diagnosed patients meet criteria for FHR MM, with median overall survival typically less than three years. Despite the clinical significance of this subgroup, few prospective trials have specifically evaluated treatment strategies for FHR disease (36, 37). Consequently, there remains an urgent need to refine risk stratification approaches and reliably identify these patients at diagnosis to enable timely intervention and risk adapted therapy.

## Recent advances in genomics and AI for redefining risk in MM

Optimal risk stratification at diagnosis increasingly depends on the comprehensive and timely integration of multimodal data, including clinical parameters, laboratory and pathology tests, cytogenetics, advanced imaging, and genomic insights derived from high-throughput platforms such as gene-expression profiling and NGS (21, 36, 38).

To collate and integrate the exponential growth of complex and diverse data, AI is increasingly relevant. AI offers a powerful means of leveraging the massive clinical datasets generated by the expansion of recently approved therapies. The pace of therapeutic advances frequently results in limited head-to-head comparisons, restricting the ability to extrapolate outcome data across regimens. This challenge is compounded by well recognized discordance in patients’ cohorts between those enrolled in clinical trials and those captured in registries or real-world cohorts. By integrating and analysing these heterogeneous data sources at scale, AI has the potential to bridge these gaps and provide more reliable, generalisable insights.

AI refers to the development of data-driven, self-operating algorithms for problem solving, with two key technologies, namely machine learning and deep learning (8, 39, 40). Machine learning uses statistical techniques, including supervised, unsupervised and reinforcement learning, to enable computer systems to learn patterns from data and adapt their behaviour based on prior experience without being explicitly programmed. Deep learning, a subset of machine learning, uses multilayered artificial neural networks to model complex patterns in data. By processing large volumes of unstructured data through these layers, deep learning mimics the human brain, learning and imitating human intelligence, supporting real-world problem solving and task performance (39, 40).

Using machine learning and deep learning, large and complex multimodal datasets in MM can be integrated and analyzed to generate insights to inform clinical decisions. AI can also enhance the predictive ability of MRD, with integration across modalities, harmonisation and automated interpretation of MRD datasets, modelling longitudinal kinetics rather than treating MRD as a binary endpoint. Analyses of phase III clinical trial datasets using mathematical modelling have identified distinct MRD kinetic patterns (e.g., depth of response, rate of clearance), to better predict progression-free survival (PFS) (41). At low MRD levels, patients exhibited either rapid disease relapse or sustained low-level residual disease. Treatment effects were most pronounced within the first 6–12 months, with strong association of relapse rates with initial residual disease burden. Model-based predictions of survival closely matched observed clinical outcomes. Together, these findings suggest that mathematical modelling of MRD kinetics may enable earlier prediction of long-term outcomes, informing adaptive therapeutic strategies (41).

However, the application and effective implementation of AI into healthcare requires training with a broad spectrum of data (sourced from trials, registries, and real-world cohorts). This would enhance their ability to generalise and apply knowledge to different scenarios.

Previous studies have shown that machine/deep learning approaches can significantly improve risk stratification in MM and other hematological malignancies, with the development of validated novel machine learning driven prognostic models (19, 38, 42–61). These include IAC-50 (46), a 50-variable machine learning model, the mmSYGNAL (43) and an AI model constructed by Maura et al. in 2024 - the Individualized Risk Model Myeloma (IRMMa) (52).

IRMMa is a predictive model derived by integrating genomic data from large clinical registries and prospective trials (52). Using deep neural networks, IRMMa incorporates demographic, clinical, genomic and therapeutic variables from 1,933 patients, stratifying them into 12 molecular subgroups with distinct prognostic profiles and therapy sensitivities. Compared with clinical staging systems such as ISS, R-ISS, and R2-ISS, IRMMa delivers a more precise, and individualized prognostic prediction and supports dynamic risk assessment based on treatment regimens. Notably, IRMMa also identifies patient subgroups likely to benefit from specific interventions such as ASCT (52).

The prognostic machine learning model developed by Park et al. (45) integrated baseline standard of care variables, with data on early treatment responses to two frontline regimens- bortezomib-melphalan-prednisone (VMP) or lenalidomide-dexamethasone (RD), to enable treatment-specific risk stratification in transplant-ineligible patients with NDMM (45).

The dynamic risk model published in 2025 by Orgueira et al. (42) analyzed a larger cohort of 10,843 patients with NDMM from the EMN-HARMONY Alliance, a consortium integrating data from trials and registries. It incorporated genomic signatures into their machine learning model to generate refined predictions of survival and progression risk, outperforming the dynamic risk prediction capabilities of ISS/R-ISS. AI-driven risk models also hold promise in the identification and stratification of FHR MM patients at diagnosis (62, 63), informing therapeutic strategy.

Collectively, these models illustrate the value of AI in complex diseases such as MM, with their capacity to leverage large, heterogeneous datasets to identify patients who may benefit from specific treatment strategies. However, several inherent limitations should be acknowledged. The accuracy and generalisability of current AI models rely heavily on the quantity and quality of data aggregated across multiple institutions. Most models generate probabilistic inferences, requiring prospective validation of their efficacy in diverse patient cohorts that reflect real-world demographic, racial and socioeconomic variation. To date, there is no universally accepted framework to evaluate the accuracy and clinical applicability of these models.

The rapid expansion of new therapies and combinations, including triplet and quadruplet regimens further highlights the importance of longitudinal data integration (64, 65). As AI models rely on pre-existing datasets, they can only recommend treatments included in their training data, which currently lack regimens incorporating the newer immune based therapies.

As AI risk algorithms increasingly integrate genomic landscapes with responses to immunotherapies such as Chimeric Antigen Receptor (CAR)-T and bi-specific T-cell engagers, important genomic determinants of resistance are likely to be uncovered, offering opportunities to predict treatment response and guide drug development.

The integration of AI into healthcare raises important ethical and medicolegal considerations, including concerns that AI might replace physicians in clinical decision-making. In reality, AI is far more likely to function as an advanced decision-support tool, augmenting rather than replacing physicians’ experience and clinical acumen. By synthesising complex datasets and generating data-driven insights on therapy, AI can support physicians in delivering more personalized patient care, whilst preserving the central role of human judgement, accountability and therapeutic relationship.

## Digital twins in MM: clinical applications

Despite strong predictive ability, current AI models lack the capacity to adapt to the dynamic evolution of MM biology, subclonal heterogeneity, and the diversity of the marrow microenvironment. DTs, an emerging technology in healthcare (66–72), cardiovascular disease (73), and oncology (9, 74–90), can optimize current models by continuous, bi-directional integration of real-time multimodal patient data, enabling prediction of treatment responses and outcome under different external conditions to optimize future decision-making.

**Figure 1:**
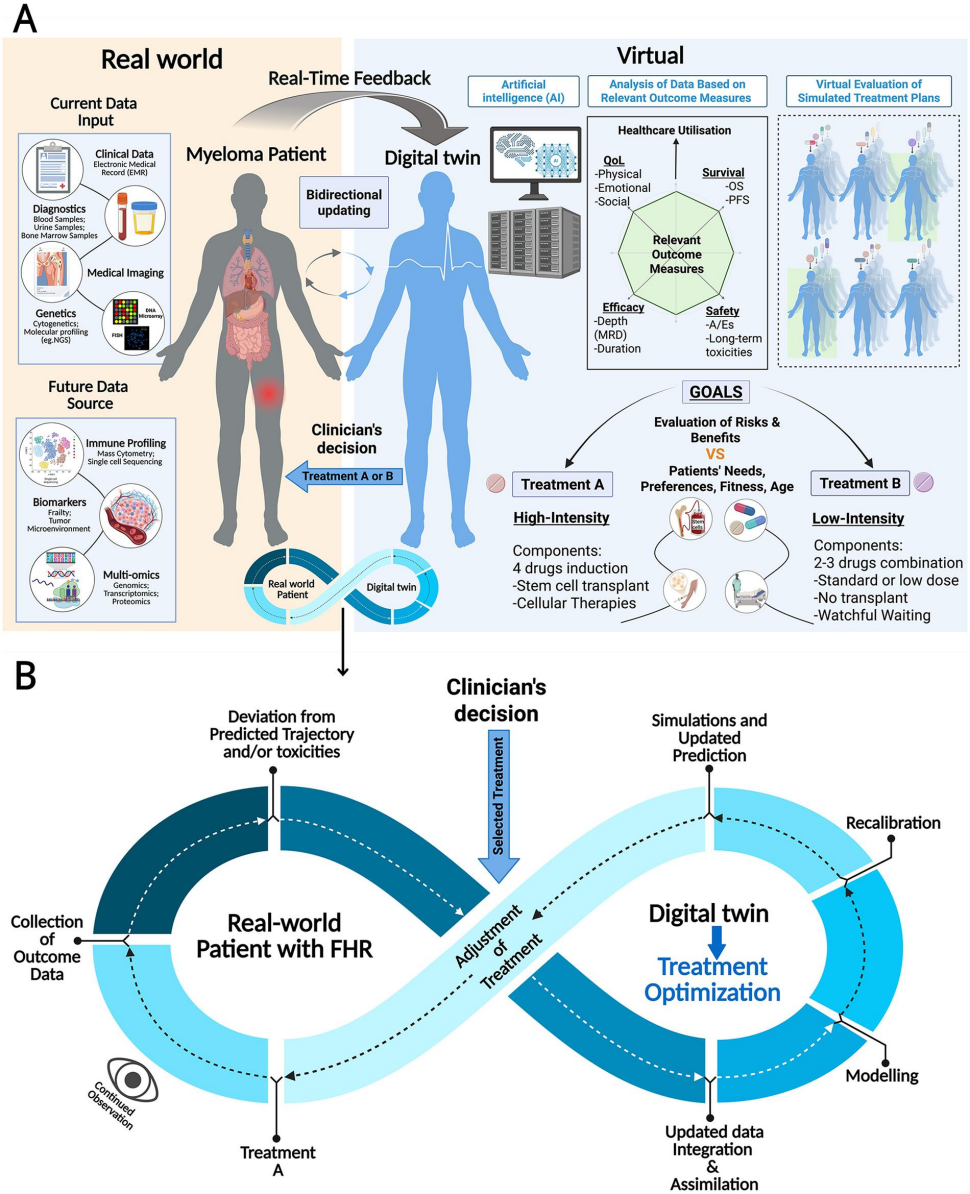
Integration of AI and digital twin technology into MM patient management. **(A)** The digital twin (DT) acts as an interactive virtual replica of the individual patient with multiple myeloma (MM), integrating multi-modal data (current and future) generated from the real-world. By modelling these data virtually to simulate outcomes, and constant updating, the DT can enable prediction of response to different treatment or intervention which can be tailored based on patient's goals and clinician's decision. This dynamic interaction ensures that the DT reflects evolving MM disease biology and treatment response. **(B)** Dynamic interaction between a real-world functional high-risk (FHR) MM patient and the DT. Longitudinal data (including outcomes, treatment response, and toxicity) are continuously collected and compared with disease trajectories predicted by the DT. Deviation from predicted response or the emergence of treatment-related toxicities prompt updated data integration and AI-driven model recalibration, with repeated simulation to refine and optimise treatment. This will enable timely adjustment of clinician-guided treatment of patients with FHR MM, enabling adaptive, personalized management. OS, overall survival; PFS, progression-free survival; NGS, next generation sequencing; A/Es, adverse events; MRD, measurable residual disease; QoL, quality of life. This figure was created in BioRender is licensed under CC BY 4.0.

In the context of MM, as shown in Figure 1, DT, a real-time, interactive, virtual replica of a patient with MM, is customized using real-world data and future longitudinal datasets (Figure 1 and Table 1). These inputs are embedded into physics-based and AI-based models capable of simulating therapeutic scenarios, forecasting disease trajectories, and directing real-time treatment decisions. Using longitudinal physiological, genomic and biomarker signature data, the DT can evolve, recalibrating risks through periodic data collection. This enables dynamic prediction of the patient's health status, transforming this into actionable decision support tools.

Through continuous, iterative integration of real-world patient data including MRD kinetics, DTs can generate individualized predictions, moving away from population-level estimates of overall response or survival. In contrast to current AI risk models for MM, DTs not only integrate multimodal data to create personalized risk prediction, but also simulate key aspects of myeloma biology, including clonal evolution, drug resistance, toxicities, and responses, across clinically relevant timepoints (post induction, post ASCT and subsequent intervals aligned with dosing schedules).

This dynamic platform informs adaptive precision therapy, enabling clinicians to rehearse, refine and compare therapeutic strategies in a virtual environment, monitor projected patient trajectories in real-time, and optimise treatment outcomes and quality of life (74).

The recent Virtual Child project (81) illustrates the transformative potential of DTs technology for malignancies such as myeloma. This initiative constructs a spatially and temporally aware *in silico* model of human development, capturing both normal and cancerous states at single-cell resolution. By generating a dynamic, adaptive, multimodal AI model of nervous system development from embryogenesis to oncogenesis, the Virtual Child project enables the simulation of unlimited virtual clinical trials, offering means to identify and prioritize treatment strategies in childhood cancer (81). MM, with its inherently complex risk stratification and persisting unpredictability in patient outcome, especially for the FHR patients despite therapeutic advances, is particularly suited to the application of DT (33, 62, 63, 83).

In patient-based applications, DTs aim to improve diagnostics, prognostication, treatment planning and overall outcomes. An optimal DT would integrate all clinically relevant variables across molecular, cellular, tissue and organ-level information (as shown in Table 1 and Figure 1), to improve the precision, accessibility and safety of treatment or interventions. As a DT is designed to mirror the MM patient in real-time, it can provide actionable insights at critical time points during a patient's journey, capturing the treatment journey in its entirety, supporting data-driven, personalized decision-making. This capability allows *in silico* evaluation of novel therapy, including optimized timing of cellular therapies such as CAR-T, bridging therapy, treatment response, risks of toxicity and adverse events, and long-term outcomes.

MRD-informed DTs, through continuous integration and analysis of multimodal data, may enable early prediction of PFS based on MRD kinetics, identify patients with rapid MRD rebound, offering the potential to identify the FHR myeloma patients earlier, to guide selection of the most effective upfront strategy (quadruplets over triplets; early incorporation of immunotherapies or treatment intensification for suboptimal responders). In addition, MRD-informed DTs may help define optimal treatment duration, including de-escalation or cessation strategies in patients with sustained MRD negativity (41).

The MM DT proposed by Grieb et al. (83) exemplifies this approach. Using a layer-based approach aligned with clinical workflows and trained on the Multiple Myeloma Research Foundation (MMRF) CoMMpass dataset, the DT simulates patient outcomes to different treatment regimens, by identifying a similarity cohort and generating probabilistic estimates of outcome (remission, time to next treatment, quality-of-life and adverse events).

In comparative simulations of two treatment regimens, Bortezomib-Lenalidomide-Dexamethasone (VRd) regimen and Bortezomib-cyclophosphamide-Dexamethasone (VCd), the DT can suggest preferences. Importantly, the tool emphasizes interpretability, transparency, and clinician oversight, flagging guideline conflicts and presenting full outcome measurements rather than recommending a single therapy, leaving the final decision with the treating clinician (83).

The CERTAINTY (A CEllulaR ImmunoTherapy VirtuAl Twin for PersonalIzed Cancer TreatmeNT) Consortium project, led by the Fraunhofer Institute for Cell Therapy and Immunology IZI, is one of the first initiatives to construct a DT for patients receiving CAR-T therapy for relapsed/refractory MM. This DT aims to reflect the patients’ individual pathophysiology and molecular patterns, and simulate the treatment journey before, during, and after CAR-T-cell infusion (79, 80). By integrating datasets, including diagnostics, single-cell multi-omics, spatial transcriptomics, and electronic health records, with mechanistic cell models, stochastic, computational structural biology, and data-centric AI approaches, the platform captures CAR-T-cell dynamics and immune interactions, predicting treatment outcome.

With continuous incorporation of new patient data, the DT also support optimisation of manufacturing processes, timing of therapy, and suggested bridging strategies. If validated, a CAR-T DT in MM could enhance clinical decision-making by anticipating toxicities, monitoring emerging resistance, and identifying bottlenecks in CAR-T production (79, 80).

## Discussion

Owing to therapeutic developments and advances in our understanding of plasma cell biology, the outcome of MM patients has dramatically improved, with refinements in prognostic models adopted in clinical practice. However, knowledge gaps persist, and MM remain incurable with heterogenous outcome, sub-optimally informed by current risk stratification models which preclude personalized therapy.

The application of AI, together with a MRD-informed DT, has potential to optimise patient outcome with a dynamic, individualized risk prediction strategy, not only to tailor therapeutic interventions, but also to accelerate development of novel treatment strategies. By simulating patients’ biological processes, responses and reactions, DTs can inform clinical research, refine treatment doses whilst minimizing risk exposure for patients. This offers tailored solutions to overcome current challenges with trials associated costs and prolonged data generation times.

In MM, novel therapies are being developed and adopted at a pace which is difficult for big data generation. Longitudinal data integration will be paramount to incorporate impact of newer anti-MM therapies, typically combined as triplets or quadruplets. In the absence of direct comparison of various multi-agent regimens, the integration of AI and DT, by leveraging multi-omics data from different sources (clinical trials, registries, real-world cohorts), represents an appealing option to inform on the optimal treatment strategy.

We are likely “missing” key prognostic information in our current AI and DT models, which limit their predictive power. While training datasets and model validation are important, these models often reinforce existing knowledge and are difficult to assess for sensitivity and specificity. A major concern is the potential exclusion of critical prognostic markers in the models, raising the question of how to adapt models as new parameters become available.

The integration of multimodal data has been discussed, but most datasets remain heavily pre-processed or constrained by current analytic techniques. Spatial and single-cell transcriptomics typically sequence only fragments of mRNA, and most DNA sequencing platforms do not capture epigenetic modifications. Additionally, short-read genomic sequencing is rarely phased, due to file-format and dataset sizes limitations, obscuring which variants reside on the same chromosome. Furthermore, most genomic data are aligned to a reference rather than analyzed *de novo*. Any of this missing information could be critical for building robust generalizable predictive models.

In practice, the integration of AI and DTs in MM faces several technical and methodological challenges, including risks of unreliable predictions, non-factual outputs, reasoning errors, systematic biases, and limited interpretability (84). Despite the biological complexity of MM, marked by clonal and spatial heterogeneity, and pronounced inter- and intra-patient variability, the integration of large-scale multimodal data poses major computational challenges. These arise not only from the sheer volume of information, but also from the diversity of data types, spanning genomics, imaging, and electronic health records, alongside additional variables such as lifestyle, physiological parameters, and environmental exposures.

Fragmentation across heterogeneous data sources, siloed genomic repositories, and non-interoperable electronic health records systems further impedes harmonization and complicates comprehensive data integration and analysis. Ensuring the relevance and applicability of the MM DT requires accurate data captured through validated protocols, standardized formats, and consistent assay methodologies to minimize data heterogeneity. High-quality data can then be integrated into reliable, real-time data processing platforms to reduce latency and support robust model performance.

To date, DT remains clinically unvalidated in MM, limiting its current application to research use. To attain regulatory approval and clinical integration into healthcare, evidence-based validation will be needed, at a level consistent with the current requirements for approval of genomic biomarker platforms (75). Further challenges in the integration of DT relate to privacy and regulatory challenges. In standardizing the applications of DT, frameworks such as the European AI Act and the European Medicines Agency's guidelines are critical, to ensure that models meet rigorous standards of accuracy, privacy, and ethical compliance, which are vital for protecting patient rights and supporting DT's trustworthiness and reliability.

## Future directions: implications for clinical practice

As we move into the era of increasingly personalized therapy, accurate risk stratification and identification of patients with the poorest outcomes, the FHR patients, is critical, not only to guide treatment, but also to prioritize drug development. Conversely, individuals assessed to have better longer-term survival may benefit from de-escalation strategies, minimising toxicities, health care costs, and preserving quality of life without compromising efficacy. Despite the explosion of multiple novel, targeted therapies, FHR MM patients remain underserved with dismal outcome, owing to ongoing knowledge gaps and limitations of current prognostic models.

The emergence of DT technology has the potential to transform the management of patients with MM, with their ability to capture tumor biology in real-time, integrating large-scale data, to inform clinical decision by simulating treatment predictions, whilst continually updating and recalibrating to deepen understanding for improved outcomes. Recent publications on the dynamic, prognostic risk models for MM highlighted their potential, with opportunities for innovative treatment approaches and accelerate drug development (43, 44, 50).

Although the future of DT applications in MM appears promising, various challenges for its integration into routine clinical practice remain. As highlighted in the previous section, issues with technical implementation, its associated costs, infrastructure, lack of interoperability and standardization challenges, ethical and privacy concerns with the need for regulatory approval pathways and legal frameworks, are foreseeable barriers (75).

To overcome these barriers, future efforts should focus on several key aspects. There is a need for robust infrastructures to support the effective integration of large-scale, multimodal data, and longitudinal clinical records (91), along with the complexity of biological modelling. Ethical and legal considerations should be addressed to ensure responsible application of DT, with mitigation strategies such as data privacy, measures to address algorithm bias and ensure fairness, transparency and trust, and establishing clear liability standards.

Lastly, the optimal integration of DT into MM care, requires robust clinical validation, ideally through an interdisciplinary approach with an assembly of willing clinical and scientific experts. The integration of DT in MM holds great promise, but requires careful attention to ethical, legal, and operational challenges. Multidisciplinary efforts, supported by evolving regulatory frameworks, are essential for ensuring responsible and effective implementation to improve MM patient outcomes.

## References

[1] Mafra A Laversanne M Marcos-Gragera R Chaves HVS McShane C Bray F The global multiple myeloma incidence and mortality burden in 2022 and predictions for 2045. J Natl Cancer Inst. (2025) 117(5):907–14. 10.1093/jnci/djae321 39658225

[2] Zorlu T Kayer MA Okumus N Ulaş T Dal MS Altuntas F. Challenges, difficulties, and delayed diagnosis of multiple myeloma. Diagnostics. (2025) 15(13):1708. 10.3390/diagnostics15131708 40647707 PMC12249263

[3] Rajkumar SV. Multiple myeloma: 2022 update on diagnosis, risk stratification, and management. Am J Hematol. (2022) 97(8):1086–107. 10.1002/ajh.26590 35560063 PMC9387011

[4] Zanwar S Rajkumar SV. Current risk stratification and staging of multiple myeloma and related clonal plasma cell disorders. Leukemia. (2025) 39(11):2610–7. 10.1038/s41375-025-02654-y 40702148 PMC12589131

[5] Herget GW Kälberer F Ihorst G Graziani G Klein L Rassner M Interdisciplinary approach to multiple myeloma – time to diagnosis and warning signs. Leuk Lymphoma. (2021) 62(4):891–8. 10.1080/10428194.2020.1849681 33225781

[6] Bumma N Dhakal B Fraser R Estrada-Merly N Anderson K Freytes CO Impact of bortezomib-based versus lenalidomide maintenance therapy on outcomes of patients with high-risk multiple myeloma. Cancer. (2023) 129(14):2179–91. 10.1002/cncr.34778 37021929 PMC10516285

[7] Rees MJ D'Agostino M Leypoldt LB Kumar S Weisel KC Gay F. Navigating high-risk and ultrahigh-risk multiple myeloma: challenges and emerging strategies. Am Soc Clin Oncol Educ Book. (2024) 44(3):e433520. 10.1200/EDBK_433520 38772002

[8] Shouval R Fein JA Savani B Mohty M Nagler A. Machine learning and artificial intelligence in haematology. Br J Haematol. (2021) 192(2):239–50. 10.1111/bjh.16915 32602593

[9] Hernandez-Boussard T Macklin P Greenspan EJ Gryshuk AL Stahlberg E Syeda-Mahmood T Digital twins for predictive oncology will be a paradigm shift for precision cancer care. Nat Med. (2021) 27(12):2065–6. 10.1038/s41591-021-01558-5 34824458 PMC9097784

[10] Durie BGM Salmon SE. A clinical staging system for multiple myeloma correlation of measured myeloma cell mass with presenting clinical features, response to treatment, and survival. Cancer. (1975) 36(3):842–54. 10.1002/1097-0142(197509)36:3<842::AID-CNCR2820360303>3.0.CO;2-U 1182674

[11] Greipp PR San Miguel J Durie BG Crowley JJ Barlogie B Bladé J International staging system for multiple myeloma. J Clin Oncol. (2005) 23(15):3412–20. 10.1200/JCO.2005.04.242 15809451

[12] Palumbo A Avet-Loiseau H Oliva S Lokhorst HM Goldschmidt H Rosinol L Revised international staging system for multiple myeloma: a report from international myeloma working group. J Clin Oncol. (2015) 33(26):2863–9. 10.1200/JCO.2015.61.2267 26240224 PMC4846284

[13] D'Agostino M Cairns DA Lahuerta JJ Wester R Bertsch U Waage A Second revision of the international staging system (R2-ISS) for overall survival in multiple myeloma: a European myeloma network (EMN) report within the HARMONY project. J Clin Oncol. (2022) 40(29):3406–18. 10.1200/JCO.21.02614 35605179

[14] Abdallah NH Binder M Rajkumar SV Greipp PT Kapoor P Dispenzieri A A simple additive staging system for newly diagnosed multiple myeloma. Blood Cancer J. (2022) 12(1):21. 10.1038/s41408-022-00611-x 35102148 PMC8803917

[15] Avet-Loiseau H Davies FE Samur MK Corre J D'Agostino M Kaiser MF International myeloma society/international myeloma working group consensus recommendations on the definition of high-risk multiple myeloma. J Clin Oncol. (2025) 43(24):2739–51. 10.1200/JCO-24-01893 40489728 PMC13445576

[16] Bladé J Beksac M Caers J Jurczyszyn A von Lilienfeld-Toal M Moreau P Extramedullary disease in multiple myeloma: a systematic literature review. Blood Cancer J. (2022) 12(3):45. 10.1038/s41408-022-00643-3 35314675 PMC8938478

[17] Bertamini L Oliva S Rota-Scalabrini D Paris L Morè S Corradini P High levels of circulating tumor plasma cells as a key hallmark of aggressive disease in transplant-eligible patients with newly diagnosed multiple myeloma. J Clin Oncol. (2022) 40(27):3120–31. 10.1200/JCO.21.01393 35666982

[18] Garcés JJ Cedena MT Puig N Burgos L Perez JJ Cordon L Circulating tumor cells for the staging of patients with newly diagnosed transplant-eligible multiple myeloma. J Clin Oncol. (2022) 40(27):3151–61. 10.1200/JCO.21.01365 35666958

[19] Guerrero C Puig N Cedena MT Goicoechea I Perez C Garcés JJ A machine learning model based on tumor and immune biomarkers to predict undetectable MRD and survival outcomes in multiple myeloma. Clin Cancer Res. (2022) 28(12):2598–609. 10.1158/1078-0432.CCR-21-3430 35063966

[20] Shang Y Chen G Liu L Pan R Li X Shen H Clinical and immunological characteristics of high-risk double-hit multiple myeloma. BMC Cancer. (2024) 24(1):1373. 10.1186/s12885-024-13124-6 39523318 PMC11552351

[21] van Beers EH van Vliet MH Kuiper R de Best L Anderson KC Chari A Prognostic validation of SKY92 and its combination with ISS in an independent cohort of patients with multiple myeloma. Clin Lymphoma Myeloma Leuk. (2017) 17(9):555–62. 10.1016/j.clml.2017.06.020 28735890

[22] Shaughnessy JD J Zhan F Burington BE Huang Y Colla S Hanamura I A validated gene expression model of high-risk multiple myeloma is defined by deregulated expression of genes mapping to chromosome 1. Blood. (2006) 109(6):2276–84. 10.1182/blood-2006-07-038430 17105813

[23] Ebert LM Vandyke K Johan MZ DeNichilo M Tan LY Myo Min KK Desmoglein-2 expression is an independent predictor of poor prognosis patients with multiple myeloma. Mol Oncol. (2022) 16(6):1221–40. 10.1002/1878-0261.13055 34245117 PMC8936512

[24] Mason MJ Schinke C Eng CLP Towfic F Gruber F Dervan A Multiple myeloma DREAM challenge reveals epigenetic regulator PHF19 as marker of aggressive disease. Leukemia. (2020) 34(7):1866–74. 10.1038/s41375-020-0742-z 32060406 PMC7326699

[25] Mian HS Visram A Shih SC Trudel S Hay AE LeBlanc R Minimal residual disease testing infrastructure in multiple myeloma: guidance for clinical trial and routine practice use in Canada. Clin Lymphoma Myeloma Leuk. (2025) 25(6):e404–10. 10.1016/j.clml.2025.01.010 39952851

[26] Chen B Pan Q Dong Y. Advancing MRD detection in multiple myeloma: technologies, applications, and future perspectives. Am J Clin Oncol. (2025) 48(7):376–80. 10.1097/COC.0000000000001197 40214184 PMC12180695

[27] Medina A Puig N Flores-Montero J Jimenez C Sarasquete ME Garcia-Alvarez M Comparison of next-generation sequencing (NGS) and next-generation flow (NGF) for minimal residual disease (MRD) assessment in multiple myeloma. Blood Cancer J. (2020) 10(10):108. 10.1038/s41408-020-00377-0 33127891 PMC7603393

[28] Zamagni E Tacchetti P Barbato S Cavo M. Role of imaging in the evaluation of minimal residual disease in multiple myeloma patients. J Clin Med. (2020) 9(11):3519. 10.3390/jcm9113519 33142671 PMC7692446

[29] Kumar S Paiva B Anderson KC Durie B Landgren O Moreau P International myeloma working group consensus criteria for response and minimal residual disease assessment in multiple myeloma. Lancet Oncol. (2016) 17(8):e328–46. 10.1016/S1470-2045(16)30206-6 27511158

[30] Messiou C Porta N Koh D-M Riddell A Downey K Croft J Whole body MRI by MY-RADS for imaging response assessment in multiple myeloma. Blood Cancer J. (2025) 15(1):122. 10.1038/s41408-025-01327-4 40675973 PMC12271311

[31] Landgren O Prior TJ Masterson T Heuck C Bueno OF Dash AB EVIDENCE meta-analysis: evaluating minimal residual disease as an intermediate clinical end point for multiple myeloma. Blood. (2024) 144(4):359–67. 10.1182/blood.2024024371 38768337 PMC11418064

[32] Bonello F Cani L D'Agostino M. Risk stratification before and during treatment in newly diagnosed multiple myeloma: from clinical trials to the real-world setting. Front Oncol. (2022) 12:830922. 10.3389/fonc.2022.830922 35356221 PMC8959380

[33] Gay F Bertuglia G Mina R. A rational approach to functional high-risk myeloma. Hematology Am Soc Hematol Educ Program. (2023) 2023(1):433–42. 10.1182/hematology.2023000443 38066896 PMC10727111

[34] Pinto V Bergantim R Caires HR Seca H Guimarães JE Vasconcelos MH. Multiple myeloma: available therapies and causes of drug resistance. Cancers (Basel). (2020) 12(2):407. 10.3390/cancers12020407 32050631 PMC7072128

[35] Lim S-l Engelhardt M Terpos E Gay F Van de Donk NWCJ Einsele H European Myeloma network consensus statement on functional high-risk multiple myeloma. Am J Hematol. (2025) 100(12):2320–32. 10.1002/ajh.70070 40926508

[36] Kaiser MF Phillip R Hall A Holroyd A Bevington L de Tute RM Ultra high-risk multiple myeloma patients with multi-hit tumours and SKY92 high risk signature are at increased risk of early relapse even when treated with extended intensified induction and consolidation - results from the Optimum/muknine trial. Blood. (2023) 142:881. 10.1182/blood-2023-177896

[37] Usmani SZ Hoering A Ailawadhi S Sexton R Lipe B Hita SF Bortezomib, lenalidomide, and dexamethasone with or without elotuzumab in patients with untreated, high-risk multiple myeloma (SWOG-1211): primary analysis of a randomised, phase 2 trial. Lancet Haematol. (2021) 8(1):e45–54. 10.1016/S2352-3026(20)30354-9 33357482 PMC8601389

[38] Chang JG Chen J Chew G-L Chng WJ. MyeVAE: a multi-modal variational autoencoder for risk profiling of newly diagnosed multiple myeloma. BMC Artif Intell. (2025) 1(1):8. 10.1186/s44398-025-00009-2

[39] Saranya A Subhashini R. A systematic review of explainable artificial intelligence models and applications: recent developments and future trends. Decis Anal J. (2023) 7:100230. 10.1016/j.dajour.2023.100230

[40] Allegra A Tonacci A Sciaccotta R Genovese S Musolino C Pioggia G Machine learning and deep learning applications in multiple myeloma diagnosis, prognosis, and treatment selection. Cancers (Basel). (2022) 14(3):606. 10.3390/cancers14030606 PMC8833500 35158874

[41] Gregory WM Prior TJ Bartlett JB Sonneveld P Dimopoulos MA Moreau P Modeling MRD changes in myeloma to understand treatment effects, predict outcomes, and investigate curative potential. Clin Cancer Res. (2025) 31(11):2154–61. 10.1158/1078-0432.CCR-24-3475 40145942 PMC12130798

[42] Orgueira A M Perez MS G D'Agostino M Cairns DA Larocca A Palacios JJL Machine learning risk stratification strategy for multiple myeloma: insights from the EMN-HARMONY alliance platform. Hemasphere. (2025) 9(10):e70228. 10.1002/hem3.70228 41080508 PMC12509237

[43] Murie C Turkarslan S Patel AP Coffey DG Becker PS Baliga NS. Individualized dynamic risk assessment and treatment selection for multiple myeloma. Br J Cancer. (2025) 132(10):922–36. 10.1038/s41416-025-02987-6 40169765 PMC12081869

[44] Hussain Z De Brouwer E Boiarsky R Setty S Gupta N Liu G Joint AI-driven event prediction and longitudinal modeling in newly diagnosed and relapsed multiple myeloma. NPJ Digit Med. (2024) 7(1):200. 10.1038/s41746-024-01189-3 39075240 PMC11286964

[45] Park SS Lee JC Byun JM Choi G Kim KH Lim S ML-based sequential analysis to assist selection between VMP and RD for newly diagnosed multiple myeloma. NPJ Precis Oncol. (2023) 7(1):46. 10.1038/s41698-023-00385-w 37210456 PMC10199943

[46] Mosquera Orgueira A González Pérez MS Díaz Arias J Antelo Rodríguez B Mateos MV. Prognostic stratification of multiple myeloma using clinicogenomic models: validation and performance analysis of the IAC-50 model. Hemasphere. (2022) 6(8):e760. 10.1097/HS9.0000000000000760 35935610 PMC9348861

[47] Mosquera Orgueira A González Pérez MS Diaz Arias J Rosiñol L Oriol A Teruel AI Unsupervised machine learning improves risk stratification in newly diagnosed multiple myeloma: an analysis of the Spanish myeloma group. Blood Cancer J. (2022) 12(4):76. 10.1038/s41408-022-00647-z 35468898 PMC9038663

[48] Mosquera Orgueira A González Pérez MS Díaz Arias J Antelo Rodríguez B Alonso Vence N Bendaña López Á Survival prediction and treatment optimization of multiple myeloma patients using machine-learning models based on clinical and gene expression data. Leukemia. (2021) 35(10):2924–35. 10.1038/s41375-021-01286-2 34007046

[49] Ferle M Grieb N Kreuz M Ader J Goldschmidt H Mai EK Predicting progression events in multiple myeloma from routine blood work. NPJ Digit Med. (2025) 8(1):231. 10.1038/s41746-025-01636-9 40307417 PMC12043975

[50] Mittelman M Israel A Oster HS Leshchinsky M Ben-Shlomo Y Kepten E Can we identify individuals at risk to develop multiple myeloma? A machine learning-based predictive model. Br J Haematol. (2025) 207(2):387–94. 10.1111/bjh.20136 40524461 PMC12378918

[51] Perrot A Lauwers-Cances V Tournay E Hulin C Chretien ML Royer B Development and validation of a cytogenetic prognostic Index predicting survival in multiple myeloma. J Clin Oncol. (2019) 37(19):1657–65. 10.1200/JCO.18.00776 31091136 PMC6804890

[52] Maura F Rajanna AR Ziccheddu B Poos AM Derkach A Maclachlan K Genomic classification and individualized prognosis in multiple myeloma. J Clin Oncol. (2024) 42(11):1229–40. 10.1200/JCO.23.01277 38194610 PMC11095887

[53] Kok CH Irani Y Clarson J Saunders V Dang P Shanmuganathan N CD302 Predicts achievement of deep molecular response in patients with chronic myeloid leukemia treated with imatinib. Blood Neoplasia. (2024) 1(2):100014. 10.1016/j.bneo.2024.100014 40454393 PMC12082111

[54] Shah MV Hung K Baranwal A Kutyna MM Al-Kali A Toop C Evidence-based risk stratification of myeloid neoplasms harboring TP53 mutations. Blood Adv. (2025) 9(13):3370–80. 10.1182/bloodadvances.2024015238 40085954 PMC12277823

[55] Kok CH Yeung DT Lu L Watkins DB Leclercq TM Dang P Gene expression signature that predicts early molecular response failure in chronic-phase CML patients on frontline imatinib. Blood Adv. (2019) 3(10):1610–21. 10.1182/bloodadvances.2019000195 31126916 PMC6538873

[56] Fitter S Bradey AL Kok CH Noll JE Wilczek VJ Venn NC CKLF And IL1B transcript levels at diagnosis are predictive of relapse in children with pre-B-cell acute lymphoblastic leukaemia. Br J Haematol. (2021) 193(1):171–5. 10.1111/bjh.17161 33620089

[57] Ansarian MA Fatahichegeni M Xu R Chen Y Wang X Ren J Emerging artificial intelligence technologies for risk assessment and management in acute myeloid leukemia: a review. JAMA Oncol. (2025) 11 (12):1518-26. 10.1001/jamaoncol.2025.3601 41196612

[58] Bernard E Tuechler H Greenberg PL Hasserjian RP Arango Ossa JE Nannya Y Molecular international prognostic scoring system for myelodysplastic syndromes. NEJM Evid. (2022) 1(7):EVIDoa2200008. 10.1056/EVIDoa2200008 38319256

[59] Irani YD Hughes A Clarson J Kok CH Shanmuganathan N White DL Successful treatment-free remission in chronic myeloid leukaemia and its association with reduced immune suppressors and increased natural killer cells. Br J Haematol. (2020) 191(3):433–41. 10.1111/bjh.16718 32352166

[60] Sharplin K Proudman W Chhetri R Tran ENH Choong J Kutyna M A personalized risk model for azacitidine outcome in myelodysplastic syndrome and other myeloid neoplasms identified by machine learning model utilizing real-world data. Cancers (Basel). (2023) 15(16):4019. 10.3390/cancers15164019 37627047 PMC10452100

[61] Montarello N Leslie A Chhetri R Friel O Singhal D Ross D Personalized risk model for predicting risk of acute coronary syndrome in patients with myelodysplastic syndromes. Blood Adv. (2023) 7(13):3032–5. 10.1182/bloodadvances.2022009173 36884290 PMC10331405

[62] Soekojo CY Chung T-H Furqan MS Chng WJ. Genomic characterization of functional high-risk multiple myeloma patients. Blood Cancer J. (2022) 12(1):24. 10.1038/s41408-021-00576-3 35102139 PMC8803925

[63] Beer SA Cairns DA Pawlyn C Holroyd A Ferris E Cook G Challenging the concept of functional high-risk myeloma through transcriptional and genetic profiling. Blood. (2025) 146(22):2670–80. 10.1182/blood.2025029987 40834881 PMC12824701

[64] Sidana S Kumar S Fraser R Estrada-Merly N Giralt S Agrawal V Impact of induction therapy with VRD versus VCD on outcomes in patients with multiple myeloma in partial response or better undergoing upfront autologous stem cell transplantation. Transplant Cell Ther. (2022) 28(2):83.e81–89. 10.1016/j.jtct.2021.10.022 PMC8900987 34781066

[65] Sonneveld P Dimopoulos MA Boccadoro M Quach H Ho PJ Beksac M Daratumumab, bortezomib, lenalidomide, and dexamethasone for multiple myeloma. N Engl J Med. (2024) 390(4):301–13. 10.1056/NEJMoa2312054 38084760

[66] Laubenbacher R Adler F An G Castiglione F Eubank S Fonseca LL Forum on immune digital twins: a meeting report. NPJ Syst Biol Appl. (2024) 10(1):19. 10.1038/s41540-024-00345-5 38365857 PMC10873299

[67] Katsoulakis E Wang Q Wu H Shahriyari L Fletcher R Liu J Digital twins for health: a scoping review. NPJ Digit Med. (2024) 7(1):77. 10.1038/s41746-024-01073-0 38519626 PMC10960047

[68] Coveney P Highfield R Stahlberg E Vázquez M. Digital twins and big AI: the future of truly individualised healthcare. NPJ Digit Med. (2025) 8(1):494. 10.1038/s41746-025-01874-x 40750826 PMC12317130

[69] Laubenbacher R Mehrad B Shmulevich I Trayanova N. Digital twins in medicine. Nat Comput Sci. (2024) 4(3):184–91. 10.1038/s43588-024-00607-6 38532133 PMC11102043

[70] Laubenbacher R Adler F An G Castiglione F Eubank S Fonseca LL Toward mechanistic medical digital twins: some use cases in immunology. Front Digit Health. (2024) 6:1349595. 10.3389/fdgth.2024.1349595 38515550 PMC10955144

[71] Sadée C Testa S Barba T Hartmann K Schuessler M Thieme A Medical digital twins: enabling precision medicine and medical artificial intelligence. Lancet Digit Health. (2025) 7(7):100864. 10.1016/j.landig.2025.02.004 40518342 PMC12412312

[72] Emmert-Streib F Cherifi H Kaski K Kauffman S Yli-Harja O. Complexity data science: a spin-off from digital twins. PNAS Nexus. (2024) 3(11):pgae456. 10.1093/pnasnexus/pgae456 39534652 PMC11555686

[73] Coorey G Figtree GA Fletcher DF Redfern J. The health digital twin: advancing precision cardiovascular medicine. Nat Rev Cardiol. (2021) 18(12):803–4. 10.1038/s41569-021-00630-4 34642446

[74] Shen S Qi W Liu X Zeng J Li S Zhu X From virtual to reality: innovative practices of digital twins in tumor therapy. J Transl Med. (2025) 23(1):348. 10.1186/s12967-025-06371-z 40108714 PMC11921680

[75] Asghar US Chung C. Application of digital twins for personalized oncology. Nat Rev Cancer. (2025) 25(11):823–5. 10.1038/s41568-025-00850-7 40634598

[76] Görtz M Brandl C Nitschke A Riediger A Stromer D Byczkowski M Digital twins for personalized treatment in uro-oncology in the era of artificial intelligence. Nat Rev Urol. (2026) 23:29–39. 10.1038/s41585-025-01096-6 41073794

[77] Wang H Arulraj T Ippolito A Popel AS. From virtual patients to digital twins in immuno-oncology: lessons learned from mechanistic quantitative systems pharmacology modeling. NPJ Digit Med. (2024) 7(1):189. 10.1038/s41746-024-01188-4 39014005 PMC11252162

[78] Lammert J Pfarr N Kuligin L Mathes S Dreyer T Modersohn L Large language models-enabled digital twins for precision medicine in rare gynecological tumors. NPJ Digit Med. (2025) 8(1):420. 10.1038/s41746-025-01810-z 40634659 PMC12241315

[79] Reiche K Weirauch U Kreuz M Fischer L Gras L Neumuth T Virtual twins for personalised CAR T-cell therapy in myeloma. Lancet Haematol. (2025) 12(7):e490–1. 10.1016/S2352-3026(25)00170-X 40610172

[80] Weirauch U Kreuz M Birkenbihl C Alb M Quaranta M Calzone L Design specifications for biomedical virtual twins in engineered adoptive cellular immunotherapies. NPJ Digit Med. (2025) 8(1):493. 10.1038/s41746-025-01809-6 40750653 PMC12316993

[81] Gilbertson RJ Behjati S Böttcher AL Bronner ME Burridge M Clausing H The virtual child. Cancer Discov. (2024) 14(4):663–8. 10.1158/2159-8290.CD-23-1500 38571421

[82] Meghdadi B Al-Holou WN Scott AJ Mittal A Liang N Sravya P Digital twins for *in vivo* metabolic flux estimations in patients with brain cancer. Cell Metab. (2026) 38(1):228–46.e17. 10.1016/j.cmet.2025.10.022 41330373 PMC12695069

[83] Grieb N Schmierer L Kim HU Strobel S Schulz C Meschke T A digital twin model for evidence-based clinical decision support in multiple myeloma treatment. Front Digit Health. (2023) 5:1324453. 10.3389/fdgth.2023.1324453 38173909 PMC10761485

[84] Bouriga R Bailleux C Gal J Chamorey E Mograbi B Hannoun-Levi JM Advances and critical aspects in cancer treatment development using digital twins. Brief Bioinform. (2025) 26(3):bbaf237. 10.1093/bib/bbaf237 40458986 PMC12130972

[85] Ștefănigă SA Cordoș AA Ivascu T Feier CVI Muntean C Stupinean CV Advancing precision oncology with digital and virtual twins: a scoping review. Cancers (Basel). (2024) 16(22):3817. 10.3390/cancers16223817 39594772 PMC11593079

[86] McCoy M. Digital twins in oncology: where we are and where we hope to go. BMJ Oncol. (2025) 4(1):e000893. 10.1136/bmjonc-2025-000893 41229611 PMC12603708

[87] Stahlberg EA Abdel-Rahman M Aguilar B Asadpoure A Beckman RA Borkon LL Exploring approaches for predictive cancer patient digital twins: opportunities for collaboration and innovation. Front Digit Health. (2022) 4:1007784. 10.3389/fdgth.2022.1007784 36274654 PMC9586248

[88] Pérez-Benito Á García-Aznar JM Gómez-Benito MJ Pérez M. Patient-specific prostate tumour growth simulation: a first step towards the digital twin. Front Physiol. (2024) 15:1421591. 10.3389/fphys.2024.1421591 39539952 PMC11557540

[89] Chang HC Gitau AM Kothapalli S Welch DR Sardiu ME McCoy MD. Understanding the need for digital twins’ data in patient advocacy and forecasting oncology. Front Artif Intell. (2023) 6:1260361. 10.3389/frai.2023.1260361 38028666 PMC10667907

[90] Karaman I Sebin B. From data-driven cities to data-driven tumors: dynamic digital twins for adaptive oncology. Front Artif Intell. (2025) 8:1624877. 10.3389/frai.2025.1624877 40785836 PMC12331622

[91] Oyetoro O. Application of artificial intelligence in predicting survival outcomes in multiple myeloma cancer patients: a systematic review. Int J Innov Sci Res Technol. (2025) 10(10):2257–63. 10.38124/ijisrt/25oct1106

